# 2157 The socially animated machine (SAM) robot: A social skills intervention for children with autism spectrum disorder

**DOI:** 10.1017/cts.2018.190

**Published:** 2018-11-21

**Authors:** Jenna Lebersfeld, Caleb J. Brasher, Christian D. Clesi, Carl E. Stevens, Fred J. Biasini, Maria I. Hopkins

**Affiliations:** University of Alabama at Birmingham

## Abstract

OBJECTIVES/SPECIFIC AIMS: Autism spectrum disorder (ASD) is a neurodevelopmental disorder that affects one in 68 children. Children with ASD have 2 core areas of difficulty: social communication skills and restricted and repetitive interests and patterns of behavior. Children with social skills deficits are at higher risk of developing mental health problems, and underdeveloped social skills predict poorer quality of life in adulthood. Therapies have been developed to help people with ASD improve social abilities in childhood, often involving a clinician directly teaching social skills lessons, either one-on-one or in a group setting. However, children with ASD can become anxious when interacting with other people and have an intrinsic motivation to interact with technology. To capitalize on this interest, this research team developed a robot, the socially animated machine (SAM) to teach social skills to children with ASD. Previous research found that this intervention was feasible and enjoyable for children with ASD and average cognitive ability, and participants improved in complex emotion recognition following intervention. The purpose of this study was to determine whether participants of all IQ levels were motivated by the SAM intervention, and whether they improved on emotion identification, facial recognition, social skills, and adaptive behavior. METHODS/STUDY POPULATION: This study recruited 20 children with ASD ages 5–14. Children completed tasks measuring ASD symptoms, IQ, receptive language, facial recognition, and emotion identification and were assigned to the control group (nonemotion dance games with SAM robot) or the intervention group (emotion games with SAM robot). Parents and teachers completed questionnaires about the child’s social skills. Following the robot intervention, facial recognition, emotion identification, and social skills were measured again, and parents and children rated participant enjoyment during the robot interaction. RESULTS/ANTICIPATED RESULTS: Overall, parents and children in both groups rated the robot interaction as highly enjoyable and motivating (parent ratings: M=26.4 out of 30, child ratings: M=17.5 out of 20). There were no differences between groups on post-test measures when controlling for pre-test scores (all *p*>0.05). Both groups improved over time on emotion identification accuracy (intervention: M=13.0% improvement, *t*=2.57, *p*<0.05; control: M=10.2% improvement, *t*=2.38, *p*<0.05) and parent-rated social skills (intervention: pre-test M=113.8, post-test M=100.6, *t*=−3.37, *p*=0.01; Control: pre-test M=107.9, post-test M=89.0, *t*=−2.83, *p*<0.05; decrease in scores indicates improvement). Teachers saw a decrease in problem behaviors for the intervention group (pre-test M=127.4, post-test M=119.6, *t*=−3.79, *p*<0.01, decrease in scores indicates improvement). DISCUSSION/SIGNIFICANCE OF IMPACT: This study shows that children with ASD and all levels of cognitive ability enjoyed and were motivated by the SAM robot intervention. This is particularly important for children with ASD who often have difficulty with attention and motivation. Children who are intrinsically motivated by the learning process will be more likely to benefit from it; therefore, continuing to pursue the methodology of robot-based interventions with this population is a worthwhile endeavor.

